# Motor unit firing pattern, synchrony and coherence in a deafferented patient

**DOI:** 10.3389/fnhum.2014.00746

**Published:** 2014-10-09

**Authors:** Annie Schmied, Robert Forget, Jean-Pierre Vedel

**Affiliations:** ^1^National Center for Scientific Research (Centre National de la Recherche Scientifique), Plasticité et Pathophysiologie du Mouvement, Institut de Neuroscience de la Timone, University Aix MarseillesMarseille, France; ^2^Faculté de Médecine, Ecole de Réadaptation, Centre de Recherche Interdisciplinaire en Réadaptation du Montréal Métropolitain, Institut de Réadaptation Gingras-Lindsay de Montréal, Université de MontréalMontréal, QC, Canada

**Keywords:** motor unit, firing rate, firing variability, synchronization, coherence, proprioception

## Abstract

The firing of spinal motoneurons (MNs) is controlled continuously by inputs from muscle, joint and skin receptors. Besides altering MN synaptic drive, the removal of these inputs is liable to alter the synaptic noise and, thus, the variability of their tonic activity. Sensory afferents, which are a major source of common and/or synchronized inputs shared by several MNs, may also contribute to the coupling in the time and frequency domains (synchrony and coherence, respectively) observed when cross-correlation and coherence analyses are applied to the discharges of MN pairs. Surprisingly, no consistent changes in firing frequency, nor in synchrony and coherence were reported to affect the activity of 3 pairs of motor units (MUs) tested in a case of sensory polyradiculoneuropathy (SPRNP), leading to an irreversible loss of large diameter sensory afferents (Farmer et al., 1993). Such a limited sample, however, precludes a definite conclusion about the actual impact that a chronic loss of muscle and cutaneous afferents may have on the firing properties of human MUs. To address this issue, the firing pattern of 92 MU pairs was analyzed at low contraction force in a case of SPRNP leading similarly to a permanent loss of proprioceptive inputs. Compared with 8 control subjects, MNs in this patient tended to discharge with slightly shorter inter-spike intervals but with greater variability. Synchronous firing tended to occur more frequently with a tighter coupling in the patient. There was no consistent change in coherence in the 15–30 Hz frequency range attributed to the MN corticospinal drive, but a greater coherence was observed below 5 Hz and between 30 and 60 Hz in the patient. The possible origins of the greater irregularity in MN tonic discharges, the tighter coupling of the synchronous firing and the changes in coherence observed in the absence of proprioceptive inputs are discussed.

## Introduction

Whether engaged in postural control or movement, the activity of spinal and supraspinal motor neurons is under the continuous control of sensory feedback provided by muscle, joint and skin receptors. In humans, stimulation of muscular and cutaneous large diameter afferents produces short-latency excitatory and/or inhibitory responses of MNs (Buller et al., 1980; Garnett and Stephens, 1980; Chalmers and Bawa, 1997; Marchand-Pauvert et al., 2000). In monkeys and humans, the effectiveness of the coupling between MNs and peripheral afferents is demonstrated by the consistent post-spike changes in EMG activity obtained through spike-triggered averaging (Flament et al., 1992; Kakuda et al., 1998; McNulty et al., 1999; McNulty and Macefield, 2001; Fallon et al., 2005; Baker et al., 2006).

Very little is known concerning the alterations that may affect MN firing pattern when cutaneous and proprioceptive feedback is lacking. During transient removal of peripheral feedback from their target muscle, MNs have been found to discharge at lower frequencies than in normal conditions (Fukushima et al., 1976; Gandevia et al., 1990; Macefield et al., 1993), in keeping with a net facilitatory contribution of peripheral afferents to the MN synaptic drive. A decrease in the net excitatory drive of proprioceptive origin may, however, be compensated for by an increase in the MN synaptic drive of cortical origin, which may eventually result in higher firing rates (Garland and Miles, 1997a). Besides suppressing part of the MN net excitatory synaptic drive, the removal of afferent feedback is liable to alter synaptic noise and, hence, the variability in MN tonic activity (Calvin and Stevens, 1968; Matthews, 1996). During transient removal of peripheral feedback from their target hand muscle, human motor axons recorded during maximal contraction were found to discharge more regularly than MUs tested under normal conditions (Gandevia et al., 1990). At submaximal contraction levels, however, a marked increase in discharge variability was reported to occur (Fukushima et al., 1976; Gandevia et al., 1990; Garland and Miles, 1997a).

As a major source of common and/or synchronized inputs shared by MNs, sensory afferents may also contribute to the coupling of the discharges of MN pairs in the time and frequency domains (synchrony and coherence, respectively). The temporal coupling observed within a few ms (short-term synchronization) or tens of ms (broad-peak synchronization) by cross-correlating MN discharges (Sears and Stagg, 1976; Kirkwood et al., 1982; Datta and Stephens, 1990; Schmied et al., 1993), and the common frequency content assessed through coherence analyses (Rosenberg et al., 1989; Farmer et al., 1993; Halliday, 2000) are interpreted as reflecting the activity of common inputs and/or inputs synchronized at the pre-motoneuronal level. Short-term or broad-peak synchronization can arise when inputs fire either stochastically or rhythmically, whereas coherence within specific frequency bands is a result of rhythmically firing inputs (Rosenberg et al., 1989; Kirkwood and Sears, 1991; Baker et al., 1999; Halliday, 2000). During steady contraction, coherence between the discharges of single MUs is often prominent between 15 and 35 Hz in arm and leg muscles (Davey et al., 1993; Farmer et al., 1993; Mills and Schubert, 1995; Salenius et al., 1997; Kim et al., 2001; Kilner et al., 2002; Semmler et al., 2004). This frequency range is similar to the beta-range oscillatory coupling observed between the electroencephalographic, or magnetoencephalographic activity of the sensorimotor cortex, and the electromyographic (EMG) activity (corticomuscular coherence) in humans (Conway et al., 1995; Brown et al., 1999; Mima and Hallett, 1999; Brown, 2000; Marsden et al., 2000; Grosse et al., 2002; Salenius and Hari, 2003). Oscillatory coupling in the beta-range can similarly be found between the EMG activities of coactivated muscles (intermuscular coherence) during static isometric contraction (Kilner et al., 1999; Grosse et al., 2002).

Clinical and experimental evidence converges in support of a central origin for the synchronous activity and coherence observed between voluntarily activated MUs, with a major contribution of corticospinal pathways (Adams et al., 1989; Powers et al., 1989; Davey et al., 1990; Datta et al., 1991; Farmer et al., 1993; Schmied et al., 1993, 1999, 2000). Indeed, with the exception of one study showing that ischemic deafferentation could reduce single MU coherence between 6 and 10 Hz (Christakos et al., 2006), transient alteration of peripheral feedback was not found to change the amount of synchrony or coherence observed between tonically firing MNs in decerebrate cats (Prather et al., 2002) or in humans (Garland and Miles, 1997b).

Data reported above were obtained during transient alteration of somesthetic inputs. Documentation concerning the adaptation of MU firing patterns and EMG activity after an irreversible loss of proprioceptive feedback is limited. In a study performed in a patient (IW) with a quasi-total loss of large diameter sensory afferents, 3 MU pairs were reported to fire with no consistent changes in frequency, synchrony or coherence compared to normal subjects (Farmer et al., 1993). Such a limited sample precluded, however, a detailed assessment of the changes which might have affected the firing pattern and the oscillatory and/or non-oscillatory synchronous activity of MUs in this patient compared to healthy subjects. In another patient (GL) affected similarly by a quasi-total loss of large diameter sensory afferents, no major change was reported to affect corticomuscular coherence in the beta-range (Patino et al., 2008), whereas, in the same patient, inter-muscular coherence in the beta-range was found to be lacking during steady contraction of finger muscles (Kilner et al., 2004). Taken together, these data suggest that during isometric contraction, group I and II sensory afferents are necessary for the synchronization of the EMG activity of synergistic MN pools (Kilner et al., 2004), but not for the coupling of motor cortex and MN activity in the beta frequency range (Patino et al., 2008), nor for the synchrony and beta range coherence observed between MU discharges within a MN pool (Farmer et al., 1993). Although IW and GL both showed a quasi-total loss of large diameter afferent fibers due to SPRNP, there were, nonetheless, some differences in the extent of deafferentation, which went from the feet up to the neck in the first case, and up to the nose in the second (Cole and Paillard, 1995), and the persistence of movement-evoked potentials in self-paced movement in the first, but not in the second case (Cole et al., 1995; Kristeva et al., 2006).

The present study aimed at investigating the influence of muscle and cutaneous afferents on the frequency and variability of single MU tonic activity, and on the coupling of the discharges of MU pairs in the time and frequency domains in the patient GL in whom corticomuscular and intermuscular coherence had previously been investigated (Kilner et al., 2004; Patino et al., 2008). To this aim, inter-spike interval, cross-correlation and coherence analyses were applied to the activity of 92 MU pairs in the wrist extensor muscles tested during low-force handgrip. Data were compared with those obtained for 171 MU pairs tested under the same conditions in 8 healthy subjects. Part of the data has been preliminarily published elsewhere (Schmied et al., 1995).

## Materials and methods

The patient GL (female) suffering from a major sensory polyradiculoneuropathy was tested at the ages of 47 and 61, in 2 sets of recordings (a, b) including 5 and 2 sessions, respectively. Data were compared with those obtained in a single session with 8 healthy female subjects of similar ages (42–63) with no signs of neurologic impairments. Experiments were conducted with the approval of the Ethics Committee of the local Medical University (CCPPRB-Marseilles I, approval No 92/74), and the informed consent of the patient and control subjects to the experimental procedure.

### Clinical description

The patient GL followed at the Centre Hospitalier de l'Université de Montréal, Pavillon Hôtel Dieu (Canada), had been suffering from a permanent and specific loss of the large peripheral myelinated sensory fibers for 15 and 29 years at the time of the first and second testing, respectively. At age 28, she first developed Guillain-Barré syndrome with motor and sensory symptoms from which she completely recovered. Then, at 32, she had another episode of polyradiculoneuropathy that affected strictly her peripheral sensory nervous system, but with no recovery. History and disease characteristics were extensively described in Forget (1986). In short, this resulted in a loss of light and crude touch, vibration perception, kinaesthesia, and position sense in her four limbs, trunk, neck and face below the nose. All tendon reflexes were absent. She can feel strong pressure, as well as pain and temperature. No sensory nerve action potentials were observed from antidromic or orthodromic stimulation of the superficial radial, median and ulnar nerves in either hand, or from the left and right sural and superficial peroneal nerves. No sensory evoked potentials could be detected in the cortical sensorimotor areas. These observations have been confirmed and proven stable for the past 30 years. A sural nerve biopsy revealed a complete loss of A-β myelinated fibers larger than 9 μm (Cooke et al., 1985; Forget and Lamarre, 1995). The motor pathways were not affected and motor nerve conduction velocities and needle EMG investigation of the muscles of the arm were normal without any clinical evidence of weakness. Although confined to a wheelchair, the patient is able to do most of her daily manual work at home and, after years of training, has completely recovered fine movements such as handwriting, but only under visual guidance.

### Experimental paradigm

The patient and control subjects were all right-handed. Experiments were performed in an adjustable armchair with the right forearm held in a cushioned groove. The distal end of the forearm was immobilized in a U-shaped device maintaining the hand in a semi-prone position, with the wrist flexed at 10°. In the rest position, subjects had their fingers passively flexed around a fixed cylinder (diameter: 4 cm; length: 10 cm) placed vertically against the palm of the hand. During the recordings, subjects closed their hand around the manipulandum and maintained the position and pressure of their fingers around it as steady as possible for the tonic discharges of 2 low-threshold MUs to be recorded for 2–3 min. The MU recordings were monitored on oscilloscopes and computer screens. On-line discrimination was performed by means of dual window discriminator units (Bak electronics) to provide the subjects with visual and auditory feedback for the 2 MU discharges.

Wrist extensor and flexor EMG activity was recorded using pairs of non-polarizable single-use surface electrodes (Ag-AgCl, 16 mm^2^, Alpine Biomed) placed 2 cm apart. Single MU discharges were simultaneously recorded in the extensor carpi radialis muscles by means of two tungsten microelectrodes (impedance 12 M?, tested at 1 kHz, Frederick Haer and Co., USA) inserted transcutaneously (1–2 cm apart), and moved in tiny steps until a stable recording was obtained. EMG and MU activities were amplified and filtered (band-pass at 30 Hz-1 kHz and 300–3000 Hz, respectively). EMG and MU signals were digitized (sampling rates of 5 and 30 KHz, respectively) and stored on a computer using an acquisition device (1401-plus) driven by Spike 2–5 software (Cambridge Electronic Design, Cambridge, UK).

**Figure 1:**
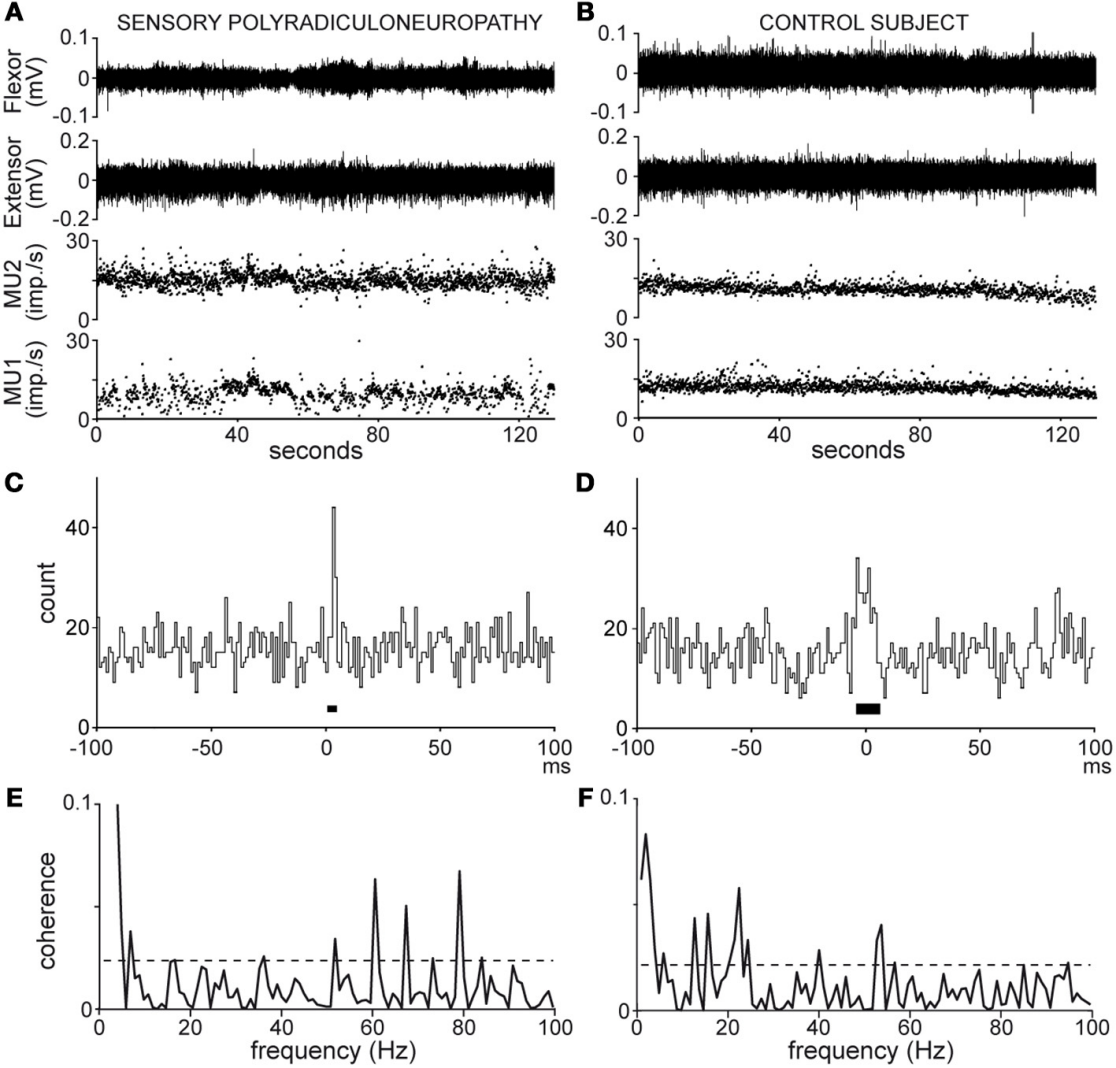
**Recordings (A,B) and analyses (C–F) of MU pair discharges in the patient (A,C,E) and healthy subject n°8 (B,D,F); (A,B), wrist flexor and extensor EMG activity (top traces), MU 1 and 2 instantaneous frequency (bottom traces); (C,D), cross-correlograms [synchronization peak width (black bar) 4 and 12 ms, synchronous impulse probability 0.04 and 0.08]; (E,F), coherence spectra (dotted line, significance limit)**.

Root mean square (RMS) values for wrist extensor and flexor EMG activity were computed across each of the recording periods. At the end of the experiment, microelectrodes were removed and subjects were asked to produce 3 bouts of maximal isometric contraction of the wrist extensor and flexor muscles, under strong verbal encouragements. The highest level of EMG activity assessed in these bouts was subsequently used to normalize EMG activity in percentages of maximal voluntary muscle contraction (% MVC). Single MU action potentials were re-discriminated off-line and analyzed using Spike 2–5 software. The firing behavior of each MU was plotted on an instantaneous frequency curve, as illustrated in Figures 1A,B (MU1, MU2, bottom traces). The presence of abnormally low or high instantaneous frequency values was carefully monitored to ensure that no spike had been missed or erroneously included in the discrimination process.

MU firing patterns were characterized on the basis of the inter-spike interval (ISI) mean duration (ISI_mean_), excluding those longer than 300 ms (about 3–4 times the mean), resulting from pauses in MU tonic activity. The discharge variability was evaluated on the basis of the ISI standard deviation (ISI_SD_) and ISI coefficient of variation (ISI_CV_) across each recording (ISI_CV_ = 100 ^*^ ISI_SD_/ISI_mean_). The firing pattern of a given MU pair was thereafter described in terms of ISI_mean_ and ISI_CV_ geometric means (ISIgeo = (ISI_mean1_^*^ ISI_mean2_)^−2^; CVgeo = (ISI_CV 1_^*^ ISI_CV 2_)^−2^).

### Analysis of MU synchronous activity

Synchronous activity in MU pairs was analyzed by cross-correlating the 2 spike trains, as shown in Figures 1C,D. The cross-correlograms yielded the distribution of impulses produced by one MU in 1 ms bins, 100 ms before and after the trigger impulses produced by the second MU, chosen as that with the lower firing rate. Synchronous impulses formed a central peak in the cross-correlograms. The peak duration (black bar around 0, Figures 1C,D) was delimited by means of the cumulative sum (CUSUM) method (Ellaway, 1978) in reference to a baseline extending from −100 to −20 ms in the cross-correlogram. In the absence of clear-cut changes in the CUSUM bin count around time 0, the strength of synchronization was arbitrarily calculated over a 20-ms window centered on 0. The synchronization strength was evaluated in terms of synchronous impulse probability (SIP) and synchronous impulse frequency (SIF). The SIP is given by the peak count above the baseline mean divided by the number of trigger spikes (i.e., imp./trig.), whereas the SIF is given by the peak count above the baseline mean divided by the duration of the recording (i.e., imp./s). The statistical significance of changes in the peak region as compared to the baseline was evaluated at *p* < 0.05 on the basis of the Z score value (*z* = 1.96) of the peak count (Garnett et al., 1976).

### Analysis of MU coherent activity

Coherence analysis provides an estimate of the frequency content and the strength of the coupling between 2 spike trains (Rosenberg et al., 1989). Coherence estimates (C) were calculated using the freeware toolbox (NeuroSpec 2.0, GNU GPL) developed in MatLab (MatWorks, Natick, MA, USA) by D. M. Halliday (University of York, York, UK). For each MU pair, analysis was performed on a number L of non-overlapping spike train segments with the following parameters:

- samp_rate = 1000 (spike train sampling rate in Hz).
- sec_tot = recording duration in s.
- seg_pwr = 10 (frequency resolution = 0.98 Hz).
- *T* = 1.024 s (segment length).
- *L* = sec_tot/T.
- opt_str = w4 (full cosine taper applied to each segment, 50% tapering at each end).
- To take into account the possible influence of the firing rates and the recording duration on synchrony and/or coherence (Bokil et al., 2007; Maris et al., 2007; Schmied and Descarreaux, 2010; Negro and Farina, 2012), 2 subsets of 45 MU pairs were selected on the basis of the similarity in their range of firing rates and analyzed over a fixed duration of 120 s.
- Coherence spectra were computed from 0 to 100 Hz as shown in Figures 1E,F. In each spectrum, a 95% confidence level (dotted lines, Figures 1E,F) was calculated [C_cl_ = 1 − (0.05)^1/L^ − 1] under the assumption that the 2 spike trains were independent (Rosenberg et al., 1989). Any coherence value reaching this level was considered to reflect significant coupling between the 2 spike trains at that frequency. The rate of occurrence of significant coherence at a given frequency (expressed as a percentage of MU pairs tested) was obtained by counting the number of pairs showing significant coherence in that bin.
- Global estimates of coherence strength were obtained for both the patient and control subjects, and subsequently compared through pooling (Amjad et al., 1997), The pooling procedure checked the homogeneity of the coherence estimates among all MU pairs within each group with an extended form of the test used to detect significant differences between two single coherence estimates (Rosenberg et al., 1989), and, provided a pooled estimate for each group. The extended difference of coherence test (Amjad et al., 1997) was used again to detect any difference between the two pooled coherence estimates which may occur beyond those detected within each pool. In the case of the subsets of 45 MUs analyzed over 120 s, significant differences in coherence were detected by subtracting the inverse arc hyperbolic tangent (tanh^−1^) of the patient subset coherence estimate from the control one (Rosenberg et al., 1989).

Coherence strength was also evaluated in terms of Z scores obtained using the Fisher transformation of the coherence inverse arc hyperbolic tangent at each frequency (*Z* = tanh^−1^(√C) ∗ 2L^−2^). An estimate of population coherence was obtained by averaging the coherence Z scores in each bin across all MU pairs tested. Coherence significance and strength were further examined within eight frequency bands (band I, 0–5 Hz; band II, 5–10 Hz; band III, 10–15 Hz; band IV, 15–30 Hz; band V, 30–45 Hz; band VI, 45–60 Hz; band VII, 60–75 Hz; band VIII, 75–90 Hz) for each MU pair. Each band includes the lower frequency limit and excluded the upper one. The rate of occurrence of significant coherence observed for N MU pairs in a given band of n bins was obtained by dividing the sum (S) of occurrences of significant coherence observed for all pairs throughout the successive bins by the product of the number of pairs and bins [S/(n^*^N)]. For each pair, an estimate of the coherence strength in each band (band Z score) was obtained by averaging the Z scores of the band's n bins. An estimate of the population coherence in each band was obtained by averaging the band Z scores of the N MU pairs.

### Statistics

Given that most of the variables assessed in the patient and the control group did not follow a normal distribution, the Wilcoxon rank sum and Kolmogoroff Smirnoff tests were used to determine statistically significant differences between the groups. The rates of occurrence of significant coherence per bin and per band observed in the patient and control subjects were compared using Fisher's exact test on an n^*^m contingency table. Statistical analyses were conducted with scripts from the MatLab statistical toolbox and central file exchange (MatWorks, Natick, MA, USA). In all tests, the level of significance was set at *P* = 0.05. Unless explicitly stated, pooled data are expressed in terms of median and inter-quartile deviation (IQD) values.

## Results

It is noteworthy that, to compensate for the loss of proprioceptive feedback, the patient had to develop a motor strategy heavily dependent upon visual feedback, as previously reported in similar conditions (Rothwell et al., 1982; Sanes et al., 1985). She had to continuously focus her attention on her hand position, as well as on the MU discharges displayed on the oscilloscope screen. Even if her ability to maintain a tonic discharge of the MUs was slightly better during the second testing, constant reliance on visual feedback remained necessary.

A total of 92 and 171 MU pairs were tested in the patient and the control subjects, respectively, at similar levels of EMG activity in the wrist extensor [median (IQD) = 8.9 (5.8) vs. (8.4) (4.4)% MVC] and flexor [6.1 (2.9) vs. 6.3 (3.9)% MVC]. There was no significant difference between groups in the recording durations [121 (83) vs. 132 (59) s, *P* = 0.2] or in the number of spikes used as triggers in the cross-correlation analyses [1229 (902) vs. 1332 (766), *P* = 0.6]. The 2 subsets of 45 MU pairs selected on the basis of their similarity in firing rates included 20 and 25 pairs in the patient recording sets a and b, and 6, 6, 3, 6, 4, 6, 6, and 8 pairs in the 8 control subjects.

### Single MU firing pattern

**Figure 2:**
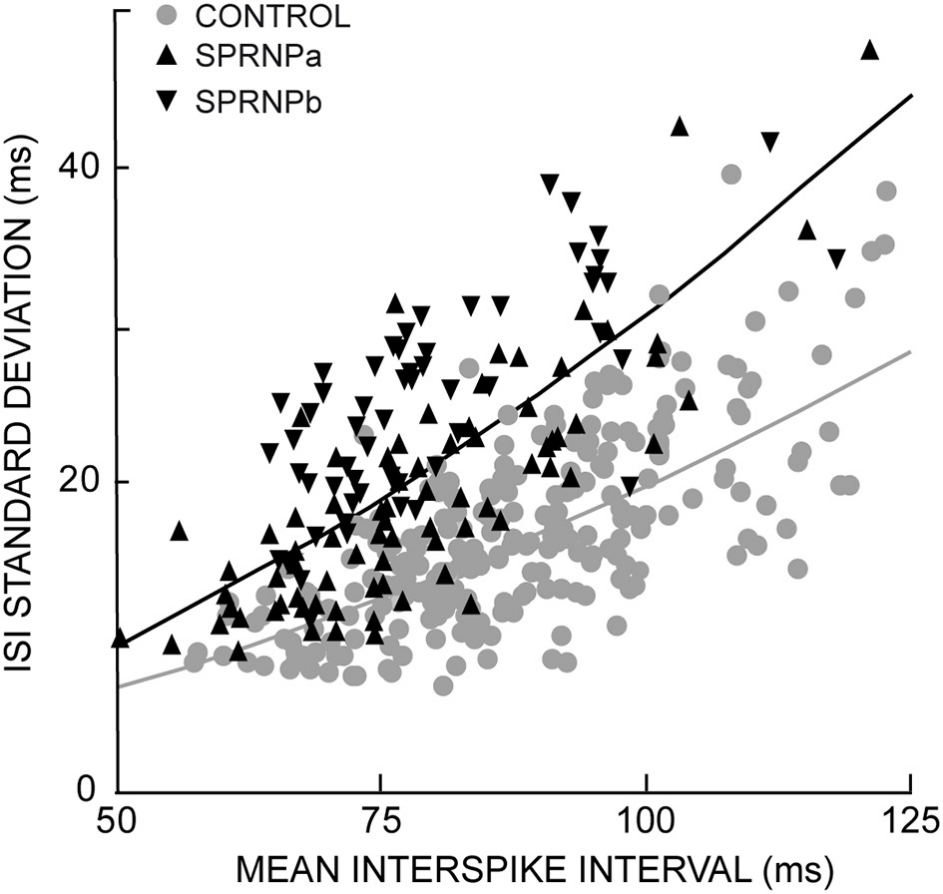
**Exponential relationship between each MU standard deviation and mean ISIs; healthy subjects gray curve and dots; SPRNP patient black curve and triangles (tip up and down, set a and b, respectively)**.

In the patient, MUs tended to fire at slightly faster rates, and greater variability than in control subjects (Table 1). The geometric means of ISI_mean_ [77.2 (17.4) ms and 87.8 (13.8) ms, respectively] and ISI_CV_ [25.7 (10.8)% and 18 (5.7)%, respectively] differed significantly between the patient and the control group (*P* < 0.0001). It is well established that MUs with faster firing rates (i.e., shorter ISI_mean_) tend to discharge more regularly (i.e., shorter ISI_SD_) than those with slower firing rates (Person and Kudina, 1972; Kukulka and Clamann, 1981; Matthews, 1996). This was confirmed in both groups. As shown in Figure 2, an exponential relationship in the form of ISI_SD_ = a^*^ISI^b^_mean_ was found in both the patient (*a* = 0.2, *b* = 1.8, goodness of fit *P* < 0.0001) and the control subjects (*a* = 0.2, *b* = 1.5, goodness of fit *P* < 0.0001). However, in both of the patient's testing sessions (SPRNPa and SPRNPb), ISI_SD_ values were consistently greater than in the control group across the whole range of ISIs (Figure 2, black triangles and gray dots, respectively). The greater firing variability of the patient's MUs as compared to control subjects was confirmed by the values of ISI_SD_ [21 (12) ms vs. 12 (5) ms, *P* < 0.0001] observed with the subsets of 45 pairs (**Table 3**) discharging with similar ISIs [80 (17) ms vs. 81 (14) ms, *P* = 0.8].

**Table 1:** **Characteristics of MU firing pattern**.

**Subject**	**Age**	***N* pairs**	**Length (s)**	**ISIgeo (ms)**	**CVgeo (%)**	**N trig**	**Synchro (%)**	**W (ms)**	**SIP (imp./trig)**	**SIF (imp./s)**
SPRNPa	47	55	101 (81)	76 (18.2)	22.1 (6.4)	1154 (993)	100	9 (5)	0.06 (0.03)	0.7 (0.3)
SPRNPb	61	37	142 (67)	78.3 (15.5)	31.6 (5.6)	1407 (825)	100	10 (3.5)	0.05 (0.02)	0.4 (0.3)
C1	42	19	170 (123)	82.9 (11)	12.4 (2.5)	2002 (1099)	74	9 (11)	0.03 (0.01)	0.3 (0.2)
C2	48	38	130 (118)	92.1 (17.8)	17.5 (3.9)	1206 (1047)	87	11.5 (5)	0.04 (0.01)	0.4 (0.1)
C3	50	9	125 (21)	92.4 (9.1)	23.1 (3.3)	1234 (390)	89	11 (7)	0.06 (0.04)	0.5 (0.4)
C4	51	18	121 (53)	78.9 (10.7)	18.7 (4.3)	1272 (757)	67	11 (10)	0.04 (0.02)	0.4 (0.2)
C5	52	19	141 (76)	96.4 (17.2)	16.7 (4.2)	1217 (891)	84	13 (6)	0.04 (0.02)	0.3 (0.2)
C6	53	26	131 (53)	88.2 (10.2)	20 (5)	1272 (701)	100	14 (8.5)	0.05 (0.03)	0.5 (0.4)
C7	57	10	128 (26)	84.9 (13.2)	20 (3)	1328 (310)	80	10 (7.5)	0.03 (0.01)	0.3 (0.2)
C8	63	32	142 (44)	83.1 (11.4)	19.1 (5.9)	1443 (585)	94	14.5 (5.5)	0.07 (0.03)	0.8 (0.3)

Recording's duration (length), geometric mean of inter-spike intervals (ISIgeo) and of its coefficient of variation (CVgeo), number of triggers (N trig), rate of significant synchrony (%), peak width (W) and strength of synchronous impulse probability (SIP) and of synchronous impulse frequency (SIF) of MU pairs in session a and b of patient GL (SPRNPa, SPRNPb), and in 8 control subjects (C1–C8).

### Synchrony between MU discharges

**Figure 3:**
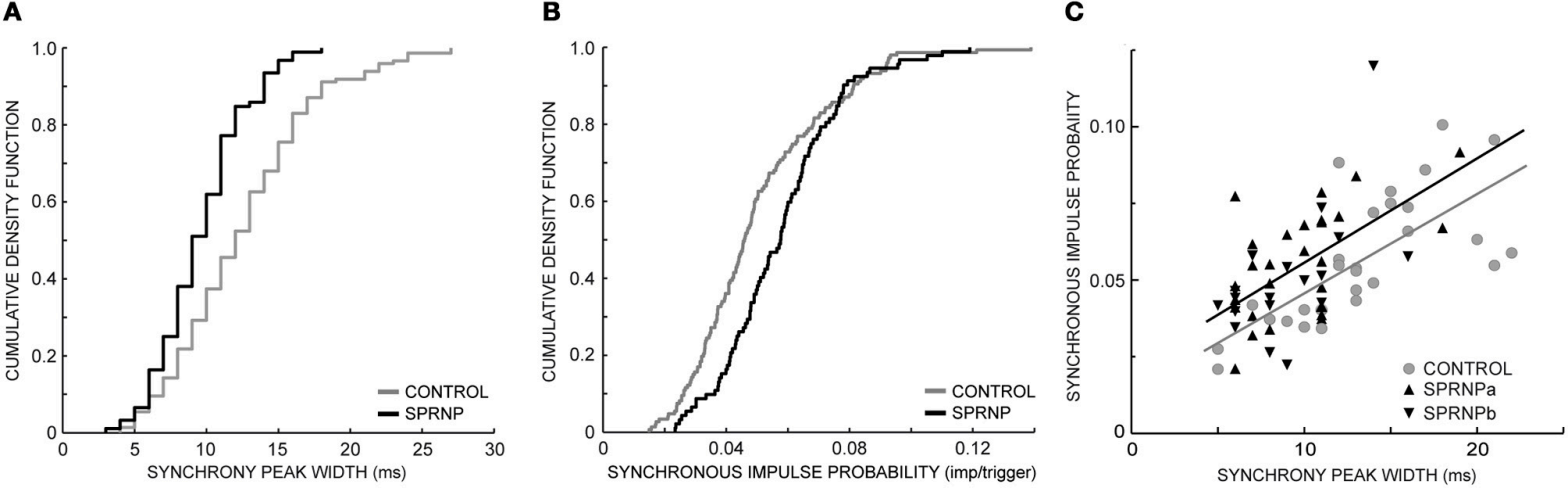
**Synchronization peak width (A) and synchronous impulse probability (B) cumulative density function (SPRNP patient and healthy subjects, black and gray staircase lines, respectively); (C) linear relationship between synchronization peak width and amplitude in two sets of 45 MUs (healthy subjects gray line and dots, SPRNP patient black line and triangles, set a tip up, and set b tip down)**.

The rate of occurrence of significant synchronization, its time course (peak width) and its strength (SIP and SIF) are summarized in Table 1 for the 2 sets of recordings performed with the patient (SPRNPa and SPRNPb) and for the 8 sessions performed with the control subjects (C1–C8). In the patient, all 92 MU pairs tested showed a significant synchronization peak whereas in the control subjects, 23 of the 171 pairs tested showed no significant synchrony. The contingency table analysis yielded a significant difference in the likelihood ratio of synchronization occurrence (*P* = 0.0001). The distribution (cumulative density function) of the significant peak width and SIP values (Figures 3A,B) also differed significantly (Kolmogoroff Smirnoff test, *P* < 0.0001 and *P* = 0.002, respectively). Upon pooling the data, the duration of significant peaks was consistently narrower in the patient than in the control group [10 (4) vs. 12 (6) ms), *P* < 0.0001]. Moreover, synchronization peaks shorter than 10 ms were observed more frequently in the patient than in the control group (48 vs. 25%, *P* < 0.0001). Synchronous firing probability and frequency indices were both found to be greater in the patient than in control subjects [SIP: 0.058 (0.027) vs. 0.046 (0.028) imp./trig., *P* = 0.0004, and SIF: 0.64 (0.38) vs. 0.45 (0.35) imp./s, *P* = 0.0005].

Comparing the subsets of 45 pairs with similar firing rates tested over 120 s confirmed that significant synchrony occurred more frequently (100 vs. 64 %) with shorter peaks [9 (4) ms vs. 13 (5) ms, *P* < 0.0001] in the patient than in the control subsets (**Table 3**). The SIP and SIF indices, however, did not differ significantly between patient and control subsets [SIP: 0.05 (0.02) vs. 0.05 (0.03) imp./trig., *P* = 0.8, and SIF: 0. 52 (0.33) vs. 0.55 (0.30) imp./s, *P* = 0.7]. There was a tendency for peaks with more bins to contain more spikes, as illustrated in Figure 3C. In both the patient and the control subsets, peak SIP values and widths were positively correlated (*r*^2^ = 0.59, *P* < 0.0001, *r*^2^ = 0.25, *P* = 0.0004, respectively) with a similar slope (0.003, *P* = 0.8) but significantly different intercepts (0.025 vs. 0.013, *P* = 0.001). Similarly, with the whole population of MUs tested, the intercept value was significantly higher in the patient than in control subjects (not illustrated), suggesting a greater effectiveness of the synchronization process.

### Coherence between MU discharges

**Figure 4:**
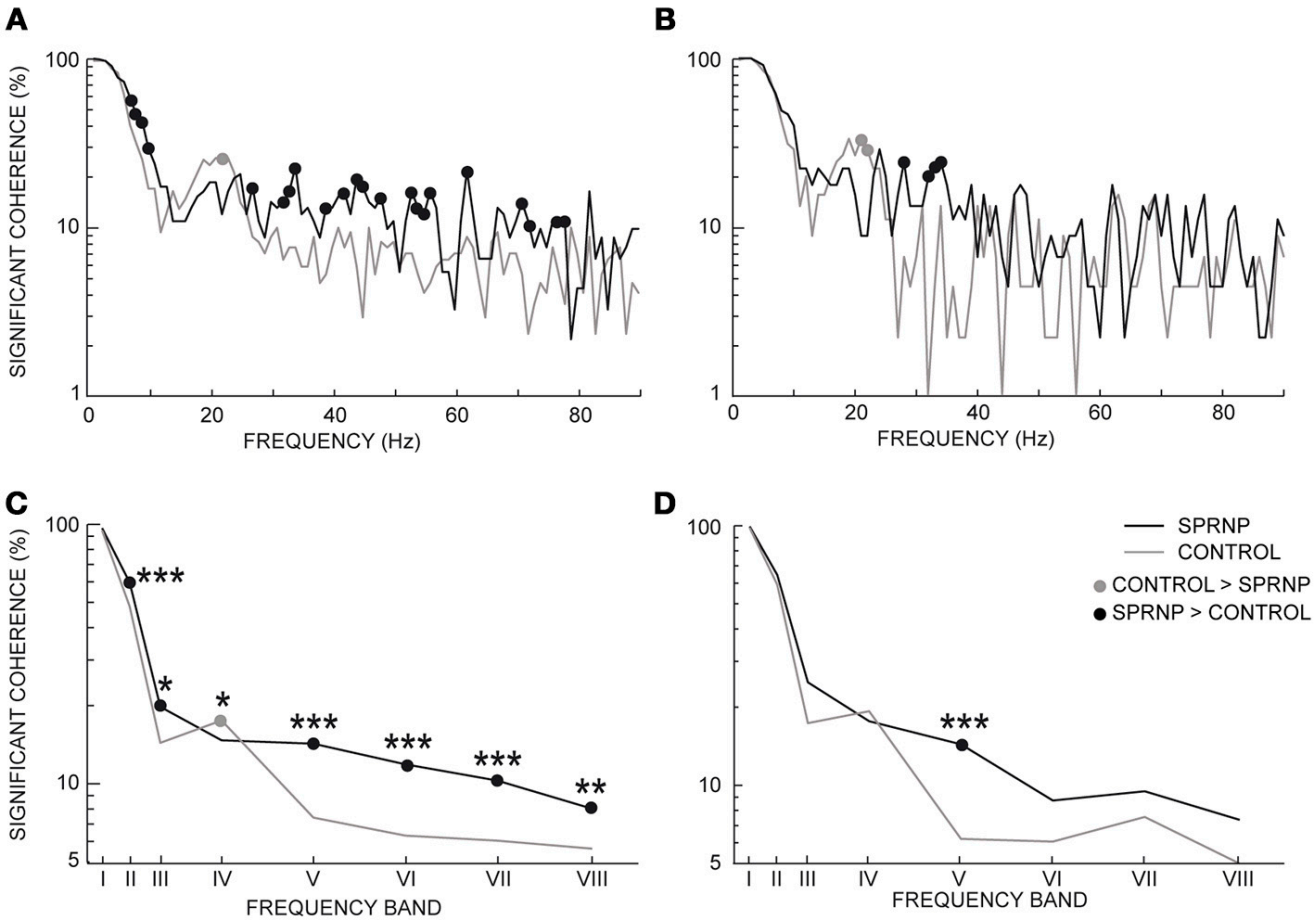
**Percentage of significant coherence values per 1 Hz-bin from all MU pairs (A), and 45 pairs firing at similar frequencies for 2 min (B); percentage of significant coherence values within 8 frequency bands for all Ms pairs (C), and the 45 pairs subsets (D); (C,D) abscissa median values of frequency bands I to VIII. (A–D)**, black and gray lines, SPRNP patient and healthy subjects, respectively; black and gray dots, significantly higher percentages in the patient and healthy subjects, respectively. ^*^p < 0.05, ^**^P < 0.01, ^***^p < 0.001.

Significant coherence values were observed more frequently in the patient than in the control subjects. This is illustrated in Figure 4 using the pooled coherence estimates computed for the whole populations of MUs tested in the patient and control group, and for the 2 subsets of 45 pairs with similar firing rates (Figures 4A,B, respectively). Applying Fisher's exact test to each bin of the spectrum with the whole MU populations revealed a significantly higher rate of significant coherence in the patient as compared to the control subjects below 10 Hz and between 27 and 79 Hz (black dots, Figure 4A), whereas the reverse was observed at 22 Hz (gray dot, Figure 4A). In the case of the subsets of 45 pairs analyzed over 120 s, the rate of occurrence of significant coherence was significantly higher in the patient than in the control group between 30 and 39 Hz (black dots, Figure 4B), whereas the reverse was observed at 21 Hz (gray dot, Figure 4B). The percentages of significant coherence and Z score values observed in each of the 8 frequency bands are summarized in Table 2 for each recording set performed with the patient (SPRNPa and SPRNPb) and for the 8 sessions with the control subjects (C1–C8). The percentages observed with the whole population of MUs tested in the patient were significantly higher than those observed for the control subjects in bands II, III, V, VI, VII, and VIII, whereas the reverse was found for band IV (Figure 4C). In the case of the subsets of 45 pairs (Table 4), the significantly higher rate of occurrence of significant coherence observed in the patient was restricted to band V (Figure 4D).

**Table 2:** **MU pairs coherence values**.

**Subject**	***N* pairs**	**Band I**	**(%, Z)**	**Band II**	**(%, Z)**	**Band III**	**(%, Z)**	**Band IV**	**(%, Z)**	**Band V**	**(%, Z)**	**Band VI**	**(%, Z)**	**Band VII**	**(%, Z)**	**Band VIII**	**(%, Z)**
SPNPa	55	94	6.2 (5)	53	2.7 (1.6)	22	1.7 (0.6)	16	1.7 (0.6)	13	1.5 (0.4)	14	1.5 (0.4)	11	1.4 (0.3)	8	1.3 (0.2)
SPNPb	37	100	9.7 (2.8)	70	3 (1.8)	17	1.4 (0.6)	12	1.5 (0.3)	16	1.6 (0.4)	9	1.5 (0.4)	10	1.3 (0.3)	8	1.3 (0.2)
C1	19	100	7.5 (3.9)	48	2.4 (1.4)	16	1.4 (0.7)	7	1.3 (0.2)	10	1.4 (0.2)	6	1.3 (0.3)	9	1.3 (0.4)	6	1.2 (0.1)
C2	38	93	7.9 (4.2)	45	2.4 (0.8)	15	1.5 (0.7)	9	1.3 (0.4)	9	1.3 (0.3)	7	1.3 (0.3)	6	1.2 (0.3)	5	1.2 (0.2)
C3	9	97	5.9 (1.9)	49	2.2 (1.7)	20	1.3 (0.8)	7	1.4 (0.3)	12	1.4 (0.4)	7	1.3 (0.3)	10	1.3 (0.3)	4	1.3 (0.2)
C4	18	86	5.2 (3.3)	38	2.1 (0.9)	8	1.4 (0.6)	8	1.3 (0.3)	4	1.3 (0.4)	7	1.3 (0.3)	6	1.3 (0.2)	5	1.2 (0.2)
C5	19	97	7.1 (3.4)	49	2.8 (1.3)	11	1.6 (0.3)	11	1.3 (0.4)	6	1.2 (0.5)	7	1.3 (0.3)	2	1.2 (0.2)	5	1.2 (0.2)
C6	26	96	7.2 (4.2)	36	2.3 (1.3)	12	1.2 (0.6)	24	1.8 (0.5)	8	1.3 (0.4)	6	1.3 (0.3)	6	1.3 (0.3)	6	1.3 (0.2)
C7	10	100	8.9 (3.7)	54	2.9 (1)	16	1.4 (1.1)	9	1.4 (0.3)	4	1.1(0.2)	3	1.3 (0.2)	7	1.3 (0.3)	6	1.3 (0.2)
C8	32	93	5.9 (2.3)	64	3 (1.1)	19	1.6 (0.8)	44	2.4 (0.7)	6	1.3 (0.2)	6	1.3 (0.3)	5	1.3 (0.3)	7	1.3 (0.2)

Rate of significant coherence (%), and coherence Z score (Z) in session a and b of patient GL (SPRNPa, SPRNPb) and in 8 control subjects (C1–C8) at each frequency band (I, 0–5 Hz; II, 5–10 Hz; III, 10–15 Hz; IV, 15–30 Hz; V, 30–45 Hz; VI, 45–60 Hz; VII, 60–75 Hz; VIII, 75–90 Hz).

**Table 3:** **Characteristics of MU subsets with similar firing range**.

**Groups**	***N***	**ISI _geo_**	**CV _geo_**	**Synchro (%)**	**W (ms)**	**SIP (imp./trig)**	**SIF (imp./s)**
SPRNPa	27	86.1 (14.8)	26.0 (9.7)	100	10 (4)	0.055 (0.03)	0.53 (0.4)
SPRNPb	18	74.3 (8.0)	29.7 (5.7)	100	8.5 (5)	0.046 (0.02)	0.52 (0.2)
CONT	45	80.2 (12.4)	15.6 (4.4)	93	12 (6)	0.040 (0.03)	0.44 (0.3)

Firing pattern (ISIgeo, CVgeo), rate of significant synchrony (%), peak width and amplitude (W, SIP, SIF) in subsets of 45 MU pairs in patient GL (SPRNPa, SPRNPb) and in control subjects (CONT).

**Table 4:** **Coherence values of MU pairs with similar firing range**.

**Groups**	***N***	**Band I (Z)**	**Band II (Z)**	**Band III (Z)**	**Band IV (Z)**	**Band V (Z)**	**Band VI (Z)**	**Band VII (Z)**	**Band VIII (Z)**
SPRNPa	27	6.6 (2.8)	2.4 (1.1)	1.5 (0.8)	1.6 (0.4)	1.5 (0.4)	1.5 (0.3)	1.3 (0.2)	1.4 (0.3)
SPRNPb	18	8.0 (1.8).	3.3 (0.8)	1.6 (0.3)	1.4 (0.5)	1.5 (0.3)	1.4 (0.2)	1.3 (0.3)	1.3 (0.2)
CONT	45	4.7 (1.5)	2.3 (0.8)	1.4 (0.5)	1.5 (0.7)	1.4 (0.3)	1.3 (0.2)	1.3 (0.2)	1.3 (0.3)

Coherence Z score (Z) in subsets of 45 MU pairs in patient GL (SPRNPa, SPRNPb) and in control subjects (CONT) at each frequency band (I, 0–5 Hz; II, 5–10 Hz; III, 10–15 Hz; IV, 15–30 Hz; V, 30-45 Hz; VI, 45–60 Hz; VII, 60–75 Hz; VIII, 75-90 Hz).

**Figure 5:**
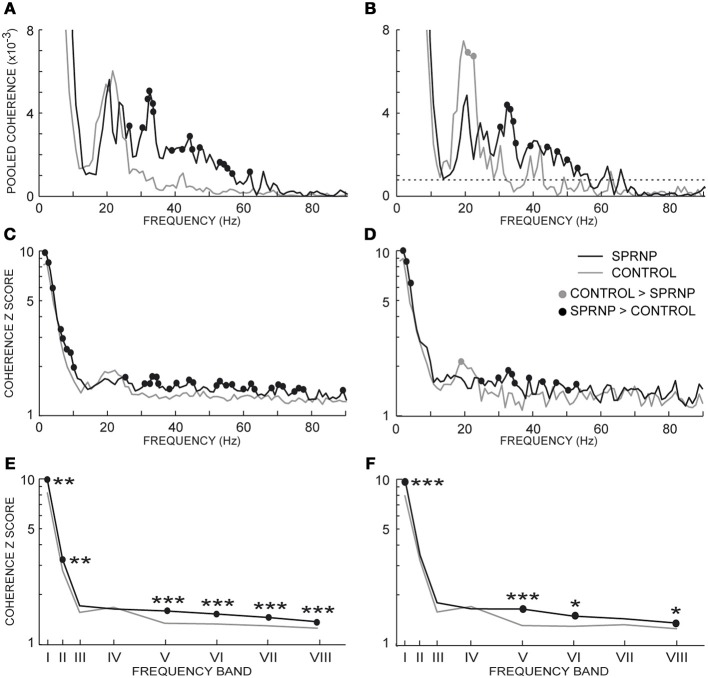
**Pooled coherence estimate spectra (1 Hz bin) for all MU pairs (A), and the subsets of 45 pairs (B, dotted line: coherence significance limit); (averaged coherence Z scores per 1 Hz-bin for all MU pairs (C), and the 45 pairs subsets (D); averaged coherence Z scores within 8 frequency bands for all MU pairs (E), and the 45 pairs subsets (F); (E,F) abscissa median values of the frequency bands I to VIII. (A–F)**, black and gray lines, SPRNP patient and healthy subjects, respectively; black and gray dots, significantly higher percentages in the patient and healthy subjects, respectively. ^*^p < 0.05, ^**^P < 0.01, ^***^p < 0.001.

Coherence estimates also tended to be stronger in the patient than in the control subjects, as seen in the whole populations of MUs tested in the patient and control subjects, and the 2 subsets of 45 pairs (Figures 5A,B, respectively). The Chi^2^ test applied to the patient pooled coherence estimate (Amjad et al., 1997) revealed significant differences between MU pairs between 1 and 9 Hz, and at 12 Hz (not illustrated), whereas in the control pool, significant differences between MU pairs occurred from 1 to 7 Hz and from 20 to 22 Hz (not illustrated). Heterogeneity in the control coherence estimate around 20 Hz is consistent with the fact that the coherence values for subjects C6 and C8 were much higher in band IV than for any of the other control subjects (Table 2). The extended Chi^2^ test applied to the mixed coherence estimate obtained by pooling together the patient and control MU recordings revealed significant differences within a region of the spectrum extending from 27 to 79 Hz (black dots, Figure 5A), i.e., well beyond the frequency regions (1–12 Hz and 20–22 Hz) where significant differences were detected within either the patient or the control pool. For the subsets of 45 pairs analyzed over 120 s, upon computing the difference between the tanh^−1^ transformation of each pooled coherence estimate (Rosenberg et al., 1989), coherence was found to be significantly stronger in the patient than in the control subjects below 5 Hz (out of scale, Figure 5B) and from 30 to 53 Hz (black dots, Figure 5B), whereas the reverse was found between 21 and 22 Hz (gray dots, Figure 5B).

Similarly, the Z scores obtained in each 1 Hz bin with all MU pairs were significantly greater in the patient than in the control subjects below 10 Hz and from 30 to 80 Hz (black dots, Figure 5C). Conversely, a non-significant trend toward greater Z scores in the control group than in the patient was observed from 18 to 22 Hz. The comparison between the 2 subsets of 45 pairs discharging at similar rates confirmed the presence of greater Z scores in the patient subset below 10 Hz and from 30 to 75 Hz (black dots Figure 5D), and the occurrence of significantly greater Z scores around 20 Hz in the control subset (gray circle Figure 5D).

The Z score means observed in the 8 frequency bands tested are shown in Table 2 for each testing in the patient and the 8 control subjects. Comparing the Z scores obtained for each band with all MUs confirmed the occurrence of stronger coherence in bands I, II, V, VI, VII, and VIII (Figure 5E) in the patient than in healthy subjects. Upon comparing the 2 subsets of 45 pairs discharging at similar rates (Table 4), coherence was also found to be stronger in the patient than in the control subsets in bands I, V, VI, and VIII (Figure 5F).

### Relationships between the MU firing pattern, synchrony and coherence indices

In order to determine whether there was a link between the changes in firing rate and variability, synchronous activity, and coherence found to affect MU discharges in the absence of sensory feedback, the relationships known to exist between some of these parameters in healthy subjects were examined in the patient. The expected positive correlation between the geometric means of ISI_CV_ and ISI_mean_ (Matthews, 1996) was observed in both the patient (ρ = 0.4, *P* < 0.0001) and the control group (ρ = 0.2, *P* = 0.03). Heterogeneity between subjects may again account for the weaker correlation observed in the control group. As reported previously in healthy subjects (Schmied and Descarreaux, 2010), there was no consistent covariation between the synchronous impulse probability (SIP) and the ISI geometric mean in both the patient (ρ = −0.05, *P* = 0.6) and the control subjects (ρ = −0.03, *P* = 0.7), whereas the synchronous impulse frequency (SIF) covaried negatively with the ISI geometric mean (ρ = −0.25, *P* = 0.001; ρ = −0.4, *P* < 0.0001, respectively) in both. Again, inter-subject heterogeneity may explain the weaker correlation in the control group. A difference appeared in the covariation commonly reported between the synchronous activity and the variability of the MU discharges (e.g., Nordstrom et al., 1992). As expected, in the control group, both the SIP and SIF values were found to increase with the geometric mean of ISI_CV_ (ρ = 0.4, *P* < 0.0001; ρ = 0.3, *P* < 0.0001, respectively). However, in the patient, the covariation was non-significant or reverted (ρ = −0.04, *P* = 0.6; ρ = −0.4, *P* = 0.0001, respectively).

The lack of sensory feedback did not seem to affect the relationship between MU firing pattern and coherence strength. As previously reported in healthy subjects (Christou et al., 2007; Schmied and Descarreaux, 2011), greater coherence values in band II (6–10 Hz) were associated with shorter ISIs (i.e., faster firing rates) in both the control and the patient MU populations (ρ = −0.3, *P* = 0.0001; ρ = −0.3, *P* = 0.002, respectively). Interestingly, in both populations, the coherence strength in band I (1–5 Hz) was found to increase with the ISI geometric mean (ρ = 0.2, *P* = 0.01; ρ = 0.6, *P* < 0.0001, respectively).

**Figure 6:**
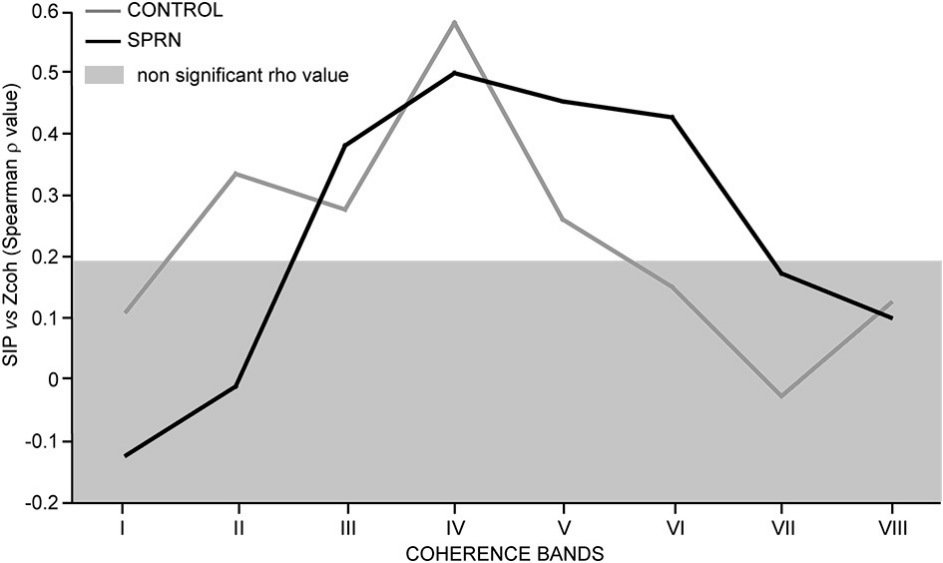
**Strength of covariation between the synchronous impulse probability (SIP) and the averaged coherence Z score (Spearman rho value, ordinate) as a function of the frequency range (bands I to VIII, abscissa); Rho values inside the light gray area are not significant; black and gray lines, SPRNP patient and healthy subjects, respectively**.

Figure 6 shows the strength of the relationships observed between the synchronous activity (SIP) and the coherence Z scores across the 8 frequency bands tested in the control and patient MU populations. In keeping with previous observations (Farmer et al., 1993; Lowery et al., 2007), the highest correlation between MU synchrony (SIP as well as SIF) and coherence was found in the beta-band IV in the control subjects. This was also seen in the patient. Again confirming previous reports (e.g., Semmler et al., 1997), the lack of correlation between the high level of coherence below 5 Hz (band I) and the amount of synchronous activity (SIP, as well as SIF) in the control group, was also observed in the patient. In the control population, a much weaker, but significant covariation was also observed in bands II, III and V. A very distinct pattern was observed in the patient, however, where the correlation was absent in band II, but particularly strong in the gamma-bands V and VI (Figure 6).

### Comparisons between the two testing sessions with the patient

Given that the testing sessions were performed at the age of 47 (set a) and 61 (set b) in the patient, it was, necessary to check whether the differences between the patient and the control subjects were present to a similar extent at each testing (cf. Tables 1–4).

The ISI geometric means assessed in sets a and b did not differ significantly, and both were significantly higher than for control subject MUs. The geometric means of ISI_CV_ were significantly greater in set b than in set a, but both were significantly higher than for control MUs.

Significant synchronization peaks were observed for all MU pairs in both recording sets. Such a high incidence of synchronization was observed only once among the 8 control subjects. The synchronization peak durations did not differ significantly between the two sessions, and were, in both cases, significantly shorter than those of the control MU pairs. Synchronization indices SIP and SIF were stronger in the first testing than in the second, but were in both cases significantly larger than those in the control group.

MU coherence spectra were remarkably similar in both recording sets, apart from the occurrence of particularly high coherence values below 5 Hz in set b. In both sets, coherence values in bands II, V, VI, and VII were significantly higher than in the control population. Moreover, the Z score values observed in bands V and VII in both of the patient recording sets were consistently greater than those observed in each of the 8 control subjects (Table 2). Both sets of recordings showed the same lack of consistent differences from the control MUs in the beta-band IV.

## Discussion

The importance of proprioceptive feedback in helping human subjects voluntarily activate single MUs, as well as the necessary role of visual and auditory feedback in the absence of muscle afferent feedback, were highlighted early on (Wagman et al., 1965; Rothwell et al., 1982; Gandevia et al., 1990). Indeed, the training required to produce and maintain steady activity in 2 MUs for 1–3 min was much longer in the patient than in control subjects. She had to visually control her arm, wrist and hand position, look at the MU spikes on the oscilloscope screen, and listen to the audio feedback, all simultaneously and continuously. The task demanded much more attention from her than from the control subjects. It is worth noting, however, that, despite the high attention load, she never complained of any form of fatigue.

In the previous case study performed with the deafferented patient IW, the limited sample of 3 MU pairs precluded a detailed assessment of the changes which might have affected the firing pattern and oscillatory and/or non-oscillatory synchronous activity of MNs, and no consistent difference was reported as compared to healthy subjects (Farmer et al., 1993). In the present study with the patient GL, the firing pattern, synchronization and coherence characteristics of single MUs were extensively documented with 55 and 37 pairs tested 15 and 29 years, respectively, after the irreversible loss of the large peripheral sensory afferents. Notwithstanding the probable existence of age-related differences in the patient's physiological state between the two recording sets, the second testing revealed the same increase in firing rate and variability, stronger short-term synchronization and greater coherence from 30 to 60 Hz as the first testing, compared to the control subjects. It is, therefore, tempting to relate the firing specificities of the MUs tested in the SPRNP patient to a putative reorganization of the MN afferent network, without excluding the possibility of adaptive changes of MN intrinsic properties facing the massive loss of synaptic inputs of sensory origin.

### Changes in firing rate

The removal of peripheral afferent feedback is liable to suppress part of the MN net excitatory synaptic drive. A marked reduction in firing rates has been observed in single motor axons and single MUs tested at maximal or submaximal contraction levels during acute deafferentation of hand or leg muscles (Fukushima et al., 1976; Gandevia et al., 1990; Macefield et al., 1993). It has been suggested that the proprioceptive feedback may contribute to up to 30% of the MN net excitatory drive (Macefield et al., 1993). Contrasting with this view a recent meta-review in which the firing rate of MNs from different muscles was analyzed in relation to the number of muscle spindles suggested that, during muscle contraction, the initial excitatory contribution of the peripheral afferent feedback may be followed by a depressing effect on the MN firing frequency (De Luca and Kline, 2012). In keeping with such a depressing effect, a trend for higher firing rates was consistently observed in the wrist extensor muscles of the chronically deafferented patient GL in both testing sessions as compared to the 8 control subjects (Table 1). A similar trend for faster firing was reported for MUs tested in healthy subjects during a postural manipulation expected to reduce muscle spindle inputs in a non-invasive way (Garland and Miles, 1997a). With the same experimental paradigm, the responsiveness of single MUs to transcranial magnetic stimulation of the motor cortex was found to be enhanced (Garland and Miles, 1997a). It was therefore suggested that an increase in the corticospinal drive could compensate for the loss of proprioceptive assistance (Garland and Miles, 1997a), in keeping with the greater excitability of the motor cortex consistently observed when sensory feedback is removed transiently (Brasil-Neto et al., 1993; McNulty et al., 2002) or after amputation (Ziemann et al., 1998). In the same way, the faster firing rates observed here in the deafferented patient may reflect a compensatory increase in corticospinal and/or subcortical MN drive allowing submaximal contractions to be sustained in the absence of proprioceptive assistance.

### Changes in firing variability

Divergent results have been reported concerning the way proprioceptive inputs may influence MN firing variability. Motor axons tested during maximum contraction were found to discharge more regularly without peripheral feedback than MUs tested under normal conditions (Gandevia et al., 1990). By contrast, MUs tested at submaximal contraction levels, were found to discharge more irregularly during pharmacological or postural manipulations expected to reduce muscle spindle input (Fukushima et al., 1976; Garland and Miles, 1997a). In the same way, the discharges of MUs tested in the deafferented patient were characterized by a much greater variability than those tested in healthy subjects. The greater irregularity of the patient's MU discharges was observed consistently in both sets recorded almost 15 years apart. Moreover, the differences between the patient and the control subjects persisted in the subsets of MUs tested over 2 min at similar firing frequencies (Table 2). The use of visual feedback has been reported to markedly enhance MU firing variability in healthy subjects older than 65 as compared to subjects below 31 (Welsh et al., 2007). In the present study, the control subjects and the patient were tested at similar ages ranging from 42 to 63. Nevertheless, the much stronger dependence of the patient on visual feedback could at least partly account for greater MU firing variability.

At the cellular level, the regularity of the discharge of a MN depends on synaptic noise and membrane properties (Calvin and Stevens, 1968; Person and Kudina, 1972; Matthews, 1996; Taylor and Enoka, 2004). Synaptic noise is liable to be altered by the suppression of the huge number of synaptic potentials normally generated by cutaneous, muscular and tendinous receptors. No prediction can be made, however, as to the extent and the sign of the putative synaptic noise changes, which may depend on the respective contribution of inhibitory and excitatory inputs still present and/or newly recruited to compensate for the loss of proprioceptive input.

Besides the likeliness of changes in the structure and pattern of synaptic noise, the intrinsic properties of the deafferented MNs may also be modified (Gonzalez-Forero et al., 2002). In a study on the effects of chronic deafferentation on cat alpha MNs, there was, however, no clear-cut evidence for changes affecting the membrane electrical properties (Gustafsson et al., 1982). In humans, a change in the after hyperpolarization duration has been reported to affect MNs in patients with amyotrophic lateral sclerosis, a neurodegenerative disease affecting both the MNs and their corticospinal afferents (Piotrkiewicz and Hausmanowa-Petrusewicz, 2011). Further investigation is required to determine if MN intrinsic properties are affected or not in the present case of deafferentation. It is noteworthy, however, that the positive correlation between the geometric means of ISI_CV_ and ISI_mean_ (Matthews, 1996) was maintained in the patient, suggesting that there was no major alteration of MN membrane properties.

### Changes in synchronous activity

The present data confirms the early observation by Stephens and colleagues in patient IW (Farmer et al., 1993) that the loss of cutaneous and proprioceptive feedback does not prevent synchronization of single MU discharges. The large sample of MUs tested here in patient GL reveals, however, the existence of changes which can be informative regarding the contribution of sensory inputs to synchronization processes in healthy subjects, as well as regarding the new synchronization patterns in the deafferented patient.

Significant peaks were observed for all MUs in both recording sets with the patient. This occurred only once in the 8 healthy subjects tested (Table 1). The most conspicuous change was the shorter duration of the synchronization peaks in the patient as compared to the control group. This was observed in both testing sets and confirmed in the subsets of MUs tested at similar firing frequencies over 120 s. The fact that peaks broader than 12 ms were less frequent in the patient suggests that proprioceptive inputs may contribute to the broad peak synchronization processes thought to involve presynaptic synchronization of MN inputs, with the contribution of segmental networks and spinal interneurons (Kirkwood et al., 1982, 1984; Powers et al., 1989; Datta et al., 1991; Schmied et al., 1994). According to data obtained in MN slice preparations (Turker and Powers, 2002), the loss of common inhibitory inputs of proprioceptive origin may also contribute to the shortening of the synchronization peaks observed in the patient.

Whether expressed in terms of SIP or SIF, synchronization tended to be stronger in the patient than in the control population, although the difference did not reach significance in the second testing. It must be kept in mind, however, that the amplitude and width of the synchronization peaks may covary (Schmied et al., 1994). As a matter of fact, in the subsets of MUs tested with similar firing rates, similar SIP and SIF values were obtained for the broad peaks with moderate bin counts observed in the control subjects and the narrow peaks with large bin counts observed in patient (Figure 3C). The higher intercept of the regression line between the peak amplitude and its duration observed in the patient reflected a trend toward greater SIP values, and, hence a greater effectiveness of the synchronization processes.

The trend toward stronger synchrony in the patient could not be accounted for by any differences in firing rate (Schmied and Descarreaux, 2010), excluded *de facto* in the subset comparisons. In addition to the strength and pattern of the synchronizing inputs, membrane properties may also influence MN synchronization (Taylor and Enoka, 2004). A putative link between membrane calcium channels, and the synchrony and the variability of the MN discharges (Taylor and Enoka, 2004) may account for the positive correlation observed between the synchrony and the variability of the MU discharges in the control group, in keeping with previous reports (Nordstrom et al., 1992; Schmied et al., 1994). In the deafferented patient, however, the increase in synchronous activity could not be attributed to the concurrent changes in the firing variability given the lack of correlation between these parameters.

The presence of large and narrow synchronization peaks in the patient suggests an enhancement of the corticospinal inputs thought to contribute to the short-term synchronization process (Datta et al., 1991). The stronger short-term synchrony observed in the patient might be related to the constant visual attention required from her to keep the same MUs discharging as steadily as possible for at least 1 min, in keeping with a previous report (Schmied et al., 2000).

Common oscillatory as well as non-oscillatory inputs are liable to generate synchronization peaks in cross-correlograms (Baker et al., 2001). Some insight into the nature of the common oscillatory inputs which may contribute to the stronger short-term synchronization observed in the deafferented patient can be gained by examining the relationship between the strength of the synchrony activity and the level of coherence in a given frequency band. In both the patient and the control populations, the strongest index of covariation was observed in a similar way in the band IV. The MN oscillatory coupling present in this frequency band (equivalent to the beta-band at the cortical level) is taken to reflect the frequency content of the corticospinal inputs which innervate MNs monosynaptically (Farmer et al., 1993, but see Mills and Schubert, 1995). Given that the synchronous activity and coherence Z score in band IV covaried similarly in the patient and the control population, it can be inferred that the loss of peripheral afferent feedback did not markedly alter the component of MU short-synchronization generated by cortical inputs firing in the beta frequency range. As a matter of fact, the major change observed in the deafferented patient was the presence of a particularly strong index of covariation between both synchrony indices (SIP as well as SIF) and the coherence Z scores in bands V and VI, contrasting with the lack of consistent covariation in the control population in this region of the spectrum (equivalent to the gamma range at the cortical level). It seems therefore that at least part of the enhanced short-synchrony observed in the patient might be explained by a greater activity of common MN inputs firing within the gamma-frequency range.

### Changes in coherence

The whole population of MUs tested in the deafferented patient showed a consistent increase in coherence below 10 Hz and above 30 Hz as compared to the control population. This was confirmed by testing 2 subsets of MUs firing at similar frequencies over 120 s in order to minimize the dependence of coherence on the duration of the spike trains (Bokil et al., 2007) and firing rates (Christou et al., 2007; Negro and Farina, 2012). Under these conditions, the most consistent changes in coherence were found in band I which includes the slow co-modulation or common drive affecting the concurrent firing of MUs (De Luca et al., 1982; Myers et al., 2004), and in bands V and VI reminiscent of the low and high gamma ranges of cortical oscillatory activity.

#### Common drive

MN coherence in the range of 1–5 Hz is correlated with the common drive index assessed by cross correlating their instantaneous firing rates (Myers et al., 2004). This low-frequency coupling represents the moment-to-moment fluctuations in the synaptic drive which controls a given set of neurons engaged in a common task. It has been observed ubiquitously in humans in MU discharges within a single muscle or in homologous bilateral muscles involved in voluntary and postural motor activity (De Luca et al., 1982; Marsden et al., 1999; Mochizuki et al., 2006), as well as in the discharges of MNs of anesthetized cats driven by cutaneous or muscle receptor afferents (Prather et al., 2002). The existence of a common drive in a muscle devoid of spindles suggests that the presence of proprioceptive afferents is not necessary for this type of coupling to occur (Kamen and De Luca, 1992). This is confirmed by the strong coherence observed within this frequency range in the deafferented patient. The enhancement of MU firing rate co-modulation in the patient is in good agreement with the stronger common drive index observed during transient suppression of proprioceptive feedback (Garland and Miles, 1997a). The enhanced low-frequency coupling between MU discharges observed in deafferented muscles is in keeping with a recent hypothesis according to which proprioceptive feedback may down-regulate the common drive (De Luca et al., 2009). Another hypothesis may also be put forth based on the motor strategy used by the deafferented patient. With respect to this, it is noteworthy that the coherence below 5 Hz was found to covary positively with the variability of the MU discharges. The major contribution of visual feedback in the deafferented patient might explain conjointly the increase in firing variability (Welsh et al., 2007) and the increase in coherence below 5 Hz (McAuley et al., 1999).

#### Beta-range coherence

Confirming data obtained in a previous study with another deafferented patient IW (Farmer et al., 1993), the range of MU coherence in the 15–30 Hz band did not differ substantially between the deafferented patient GL and the control subjects. The MUs of patient GL as well as of 6 of the control subjects did not show any conspicuous coherence peaks, in contrast to the 2 other healthy subjects. The rather moderate values of coherence observed in the beta-range frequency are in keeping with previous reports in the wrist extensor muscles (Kakuda et al., 1999; Mattei et al., 2003). The 15–30 Hz MU coherence, which is particularly prominent in finger muscles as compared to other muscles (Kim et al., 2001), is thought to reflect the frequency content of MN corticospinal inputs (Farmer et al., 1993; Moritz et al., 2005). A tight correlation between the synchrony indices and the beta-range coherence was similarly observed in the control subjects and the patient, suggesting that the lack of sensory feedback did not alter the prominent contribution of beta-range oscillatory inputs to the MU synchronous activity. In addition to the descending corticospinal drive, sensory ascending pathways have recently been shown to contribute to the beta-range oscillatory coupling of the motor cortex and MN pools with a variable degree of prominence between subjects (Riddle and Baker, 2005; Witham et al., 2011). The lack of conspicuous changes in beta-range coherence observed here at the single MU level in the deafferented patient GL fits well with the persistence of corticomuscular coherence in the beta-range described in the same patient (Patino et al., 2008). This suggests that the contribution of ascending pathways connected to the large diameter sensory afferents is not needed for this type of coupling to occur.

By contrast, the beta-range intermuscular coherence which is thought to reflect at least in part MN corticospinal inputs (Norton and Gorassini, 2006; Fisher et al., 2012) was lacking in the same patient GL (Kilner et al., 2004). This suggests a major contribution of large diameter sensory inputs shared by synergistic MN pools to this type of coupling, in keeping with data obtained in monkeys (Baker et al., 2006). It can therefore be inferred that the common inputs which generate intermuscular coherence in the beta-range frequencies differ at least partly from those which generate MU coherent activity within a muscle, and from those which generate corticomuscular coherence in the same frequency range, in keeping with previous observations (Boonstra et al., 2009; Nishimura et al., 2009; Muthukumaraswamy, 2011).

#### Gamma-range coherence

In healthy subjects, coherence between single MU discharges tested during steady isometric contractions was non-significant or very low in the gamma range as compared to the beta range frequencies, in keeping with previous studies (Davey et al., 1993; Farmer et al., 1993; Kakuda et al., 1999; Marsden et al., 1999; Kim et al., 2001; Kilner et al., 2002; Semmler et al., 2002; Mattei et al., 2003). By contrast, in the deafferented patient GL, significant coherence values were consistently observed from 30 to 60 Hz, with a greater rate of occurrence and stronger values than in healthy subjects. The occurrence of such changes was not documented in the previous study based on 3 pairs of finger muscle MUs tested in the deafferented patient IW (Farmer et al., 1993). In the deafferented patient GL, the most conspicuous enhancement of coupling between MU firings was observed in the low gamma range frequencies (30–45 Hz).

Without excluding the possible contribution of the subcortical oscillatory network which may be enhanced in the gamma-range to compensate for the loss of MN drive (Nishimura et al., 2009), one may reasonably assume that at least part of the coherent activity of single MUs originates from motor cortical areas. Corticomuscular coherence, as well as oscillatory activity in the sensorimotor cortex, has been shown to be specifically enhanced in the low-gamma range in relation to the visuomotor context and/or the degree of attention, readiness, and motor preparation in humans (Aoki et al., 1999; Schoffelen et al., 2005, 2011). An enhancement of the oscillatory corticospinal drive in the gamma-frequency range might be expected to occur in a prominent way in the deafferented patient as a result of the greater concentration and visual attention she had to develop to maintain the steady activity of pairs of MUs in the absence of peripheral feedback. This could account for the greater coupling between MU discharges observed here in the gamma frequency range.

In this context, it seems puzzling that in the same deafferented patient GL, corticomuscular coherence in the gamma-range assessed in isometric conditions did not differ from that of healthy subjects (Patino et al., 2008). The two studies differ, however, with regard to the task and muscles involved, i.e., self-adjusted hand clenching in the case of our MU coherence assessment (proactive task) vs. finger flexion in response to an external force, in the case of the corticomuscular coherence assessment (reactive task). A stronger dependence on proprioceptive feedback might be expected when the subject has to counteract an external force rather than produce the amount of force required to keep two MUs firing. Methodological differences may also contribute to the apparent discrepancy between the present single MU study and the previous corticomuscular study (Patino et al., 2008) performed in the same deafferented patient GL. Corticomuscular coherence in the beta as well as in the gamma range is known to depend on the subject's training state (Witham et al., 2011; Mendez-Balbuena et al., 2012). In the corticomuscular coherence study, a single testing session was performed with each of the 6 healthy subjects, in the same way as with the patient (Patino et al., 2008). By contrast, in the present study, MU recordings were obtained in a single session with each of the 8 healthy subjects tested, whereas 2 sets of 5 and 2 recording sessions, respectively, were performed with the patient. The longer training period and the greater effort required from the patient to keep the MU tonic discharges steady, as compared to healthy subjects, may have contributed to the stronger coupling of the MU firings in the gamma frequency ranges. It is worth noting, that, in Patino and colleagues' study, only 2 of the 6 healthy subjects tested showed significant corticomuscular coherence between 30 and 45 Hz in static conditions, while a significant peak around 35 Hz was present in the patient GL (Patino et al., 2008). This is quite consistent with the high level of MU gamma-range coherent activity presently observed in the same patient.

## Conclusion

The irreversible loss of the large diameter sensory axons which continuously convey cutaneous, muscular and tendinous feedback from the whole body is liable to impact motor control in at least two ways. Firstly, the massive loss of the peripheral afferents which control the firing of mono- and/or poly-synaptic MNs at the segmental level and/or via cortical and subcortical motor pathways must be compensated for. Secondly, new motor strategies relying on continuous visual feedback and constant attention toward the on-going motor task must be developed to replace the missing proprioceptive assistance. These two aspects must be taken into account when explaining the changes observed here in the firing patterns of single MUs, and the coupling of their discharges in the time and frequency domains. Indeed, a compensatory enhancement of corticospinal and/or descending pathways may account for the faster firing rates, greater variability, stronger short-term synchronization, and stronger coherence in the low gamma frequency range of the MUs tested in the deafferented patient. The disappearance of broad peak synchronization in the patient suggests that peripheral afferents may contribute to this type of coupling. The persistence of coherent MU activity in the beta range frequency suggests, however, that peripheral feedback is not necessary for this type of coupling to occur in wrist extensor MNs. Furthermore, the constant attention as well as the permanent reliance on visual feedback developed by the patient to cope with the loss of proprioceptive and cutaneous assistance may at least partially account for the greater firing variability, the stronger oscillatory coupling observed below 5 Hz, in the common drive frequency range, and between 30 and 60 Hz, in the gamma-frequency range.

### Conflict of interest statement

The authors declare that the research was conducted in the absence of any commercial or financial relationships that could be construed as a potential conflict of interest.
